# Cytochrome P450 Oxidoreductase Influences CYP2B6 Activity in Cyclophosphamide Bioactivation

**DOI:** 10.1371/journal.pone.0141979

**Published:** 2015-11-06

**Authors:** Ibrahim El-Serafi, Parvaneh Afsharian, Ali Moshfegh, Moustapha Hassan, Ylva Terelius

**Affiliations:** 1 Experimental Cancer Medicine (ECM), Department of Laboratory Medicine, Karolinska Institutet, Huddinge, Stockholm, Sweden; 2 Cancer Center of Karolinska (CCK), Department of Oncology-Pathology, Karolinska Institutet, Solna, Stockholm, Sweden; 3 Department of Clinical Research Centre, Karolinska University Hospital-Huddinge, Stockholm, Sweden; 4 Department of Discovery Research, Medivir AB, Huddinge, Sweden; Institute of Zoology, Chinese Academy of Sciences, CHINA

## Abstract

**Introduction:**

Cyclophosphamide is commonly used as an important component in conditioning prior to hematopoietic stem cell transplantation, a curative treatment for several hematological diseases. Cyclophosphamide is a prodrug activated mainly by cytochrome P450 2B6 (CYP2B6) in the liver. A high degree of inter- and intra-individual variation in cyclophosphamide kinetics has been reported in several studies.

**Materials and Methods:**

Hydroxylation of cyclophosphamide was investigated *in vitro* using three microsomal batches of CYP2B6*1 with different ratios of POR/CYP expression levels. Twenty patients undergoing hematopoietic stem cell transplantation were also included in the study. All patients received an i.v. infusion of cyclophosphamide (60 mg/kg/day, for two days) as a part of their conditioning. Blood samples were collected from each patient before cyclophosphamide infusion, 6 h after the first dose and before and 6 h after the second dose. *POR* gene expression was measured by mRNA analysis and the pharmacokinetics of cyclophosphamide and its active metabolite were determined.

**Results:**

A strong correlation between the *in vitro* intrinsic clearance of cyclophosphamide and the POR/CYP ratio was found. The apparent *K*_*m*_ for CYP2B6.1 was almost constant (3-4 mM), while the CL_int_ values were proportional to the POR/CYP ratio (3-34 μL/min/nmol CYP). In patients, the average expression of the *POR* gene in blood was significantly (*P* <0.001) up-regulated after cyclophosphamide infusion, with high inter-individual variations and significant correlation with the concentration ratio of the active metabolite 4-hydroxy-cyclophosphamide/cyclophosphamide. Nine patients were carriers for POR*28; four patients had relatively high *POR* expression.

**Conclusions:**

This investigation shows for the first time that *POR* besides *CYP2B6* can influence cyclophosphamide metabolism. Our results indicate that not only CYPs are important, but also *POR* expression and/or activity may influence cyclophosphamide bioactivation, affecting therapeutic efficacy and treatment related toxicity and hence on clinical outcome. Thus, both POR and CYP genotype and expression levels may have to be taken into account when personalizing treatment schedules to achieve optimal therapeutic drug plasma concentrations of cyclophosphamide.

## Introduction

Hematopoietic stem cell transplantation (HSCT) is a curative treatment strategy for malignancies, such as leukemia and lymphomas, and nonmalignant disorders such as metabolic disorders and aplastic anemia. Prior to HSCT, chemotherapy with or without radiation is used as a conditioning regimen to eliminate malignant cells, provide space for donor cells, and avoid graft rejection by suppressing the immune system [1]. Cyclophosphamide (Cy) in combination with busulphan or with total body irradiation (TBI) belong to the most common used conditioning regimens [2]. Cyclophosphamide is an alkylating agent that is widely used in the treatment of hematological malignancies as well as solid tumors. Cy is also a potent immuno-suppressive agent that affects both T- and B-lymphocytes, thus having effect on both humoral and cell-mediated immunity. Cy can also be used to treat rheumatoid arthritis, systemic lupus erythematosus, Sjögren´s syndrome, glomerulonephritis, multiple sclerosis, and in preconditioning of hosts to prevent transplant rejection [3]. Cy is a prodrug that is metabolized by several CYPs to become cytotoxic and effective in cancer chemotherapy [4]. The 4-hydroxylation product is the active metabolite of Cy that yields an active alkylating agent, phosphoramide mustard and a toxic by-product, acrolein. Acrolein is responsible for urotoxicity (hemorrhagic cystitis). Through another pathway, *N*-dechloroethylation, Cy is metabolized to an inactive metabolite (2-dechloroethyl-Cy) and the neurotoxic agent chloroacetaldehyde (CA) [5].

Cy is metabolized to the active metabolite, 4-hydroxy-cyclophosphamide (4-OH-Cy), mainly by CYP2B6 with contributions from some other CYPs. About 10% of the Cy dose is metabolized through the *N*-dechloroethylation pathway [5–7].

Several studies have shown high inter-individual variation in Cy kinetics including elimination half-life and clearance [5, 8]. Moreover, investigators have shown high inter- individual variation in expression and catalytic activity of CYP2B6 that is probably caused by the genetic polymorphism of this enzyme [9–11] or its regulation. In total, more than fifty different alleles containing point mutations have been identified to date (28-Nov-2013) (http://www.cypalleles.ki.se/cyp2b6.htm). Most of these mutations are silent but five of them result in amino acid substitutions in exons 1, 4, 5 and 9 [12].

Some of the common non-synonymous polymorphisms are reported to affect Cy kinetics either by decreased liver protein expression [12, 13] or altered function [14, 15]. In addition, several more rare non-synonymous SNPs have been found to result in the protein being absent or non-functional [16].

On the other hand, several other studies have reported that CYP2B6 genetic polymorphism doesn’t alter the Cy metabolism or the 4-OH-Cy formation *in vivo* or *in vitro* [17–19]. Moreover, Yao *et al*. suggested that clinical factors like patient age and cancer grade may contribute to the inter-individual variation in Cy kinetics [20].

Cytochrome P450 oxidoreductase (POR) is a membrane-bound enzyme in the endoplasmic reticulum that is involved in the electron transfer from the co-factor, NADPH, to CYP. POR influences the metabolic rate differently for different CYP alleles, as reported by Zhang et al [21]. POR is important in the metabolism of drugs and xenobiotics, and allelic variants and variability in expression may have clinical implications [22, 23].

POR deficiency may lead to serious complications such as disordered steroidogenesis, abnormal genitalia, bone abnormalities and William’s syndrome [24–26]. Recently, POR variants have been found to play an important role in CYP dependent metabolism of drugs and xenobiotics, at least *in vitro*. Some POR polymorphisms affect the amount of enzyme produced, which may affect the activities of cytochrome P450 enzymes such as CYP1A2, 2C9, 2C19, 2D6, 3A4 and 3A5, [27–30], especially for compounds which are rapidly metabolized and where the electron transfer from NADPH via POR may be rate-limiting.

POR variants have altered the CYP2B6 dependent activity in bupropion metabolism and *S*-Mephenytoin *N*-demethylation [31, 32]. POR*28 is the only polymorphism reported to increase CYP activity *in vivo* [33]. Many other polymorphisms are known to decrease its activity *in vivo*, for example POR*2, *3*, *4 and 5* (http://www.cypalleles.ki.se/por.htm, 11-Oct-2011).

In the present study, we aimed to investigate the effect of POR levels on the cyclophosphamide 4-hydroxylation rate *in vitro* using microsomes containing CYP2B6.1. The effect of Cy treatment on *POR* gene expression *in vivo* in patients, prior to hematopoietic stem cell transplantation (HSCT), was also investigated.

## Materials and Methods

### Chemicals

Cyclophosphamide monohydrate and *β*-Nicotinamide adenine dinucleotide phosphate reduced form (*β*-NADPH) were purchased from Sigma Aldrich (Stockholm, Sweden). Maphosphamide was used to produce 4-OH-Cy by hydroxylation in water. Maphosphamide was kindly provided by Professor Ulf Niemeyer, Baxter Oncology GmbH (Frankfurt am Main, Germany). Maphosphamide (Asta-Z 7557; MW 500.4) is a stable form for 4-OH-Cy (4-OH-Cy connected to cyclohexylamine), that is suitable for *ex vivo* experiments. The compound hydrolyses spontaneously in aqueous solution producing 4-OH-Cy [34, 35].

Potassium phosphate buffer (50 mM, pH 7.4) was used for the microsomal incubations. Acetonitrile, phosphate salts, phosphoric acid, other chemicals and solvents used were of high performance liquid chromatography (HPLC) or analytical grade and were purchased from Merck, Germany.

QuickPrep Total RNA Extraction Kit (GE Life Sciences, Uppsala, Sweden), TaqMan Reverse Transcriptase-complementary DNA (RT-cDNA) Kit (Applied Biosystems, Roche, NJ, USA), and NimbleGen microarrays (Roche Diagnostics Scandinavia, Bromma, Sweden) were used for gene array experiments. TaqMan genotyping polymerase chain reaction (PCR) primer for POR*28 SNP (rs1057868C>T, catalogue #4362691) was purchased from Applied Biosystems (Stockholm, Sweden) while the genotyping master mix (catalogue # 208252) was from Qiagen (Stockholm Sweden).

### Microsomes

Commercially available microsomes, containing human CYP2B6.1 and human POR coexpressed in *Escherichia coli* were purchased from Cypex Ltd. (Dundee, UK). Batches were available with up to approximately 18-fold differences in POR/CYP ratios (Table 1). Microsomes containing only human POR (without CYPs) from the same vendor have been used as controls (Table 1). The POR activities reported in the data sheets from the vendor were used.

**Table 1:** Characteristics of commercial microsomes containing recombinant human CYP2B6.1 and POR.

Batch No.	Batch 1	Batch 2	Batch 3	POR
**Catalogue No.**	CYP/EZ016	CYP/EZ041*	CYP020	CYP004
**Lot. No**	C2B6LR002/A	C2B6BR016A	C2B6R005	RED004
**Vendor Company**	Cypex	Cypex	Cypex	Cypex
**P450 concentration** **	1	1	1	N/A
**Protein concentration** ***	10	10	10.6	12.7
**Specific P450 content** ^**#**^	100	100	94	N/A
**Cytochrome c reductase activity** ^**##**^	77	890	1302	2437

Batch 1–3 were microsomes (Bactosomes) from *E*. *Coli* containing co-expressed CYP2B6*1 and human *POR*. The POR batch was a control without CYP.

*Batch 2 also contained a supplement of purified human cytochrome b5

**P450 concentration (nmol/mL)

***Protein concentration (mg/mL)

^#^Specific P450 content (pmol/mg protein)

^##^Cytochrome c reductase activity (nmol/min/mg protein) as marker for POR activity

N/A Not applicable

### *In vitro* cyclophosphamide metabolism assays

The effect of different POR/CYP ratios on cyclophosphamide metabolism was studied.

Cy metabolism was studied with both time and CYP concentration, and the 4-OH-Cy formation was found to be linear up to 1 mM Cy and at CYP concentrations between 10 and 40 pmol/mL and incubation times up to 25 min. Enzyme kinetic parameters were determined under linear conditions.

Three batches of cDNA-expressed human CYP2B6.1 with similar specific CYP concentrations but different POR concentrations and thus POR/CYP ratios (Table 1) were incubated for 15 min in two independent experiments at 37°C, in a total volume of 100 μL, containing 50 mM potassium phosphate buffer, pH 7.4, and 1 mM NADPH. The reactions were stopped with 100 μL of ice-cold acetonitrile. The mixtures were vortexed and centrifuged at 3000 *g* for 5 min to remove precipitated protein. Negative control incubations were run in parallel without cyclophosphamide, without NADPH, or with control yeast microsomes not expressing the human enzymes. Microsomes containing POR only, were incubated as controls in parallel, under the same conditions, after adjusting the POR amount to be equal to that of the CYP2B6.1 batch with the highest POR content.

For the determination of apparent *K*_*m*_ and *V*_*max*_, nine substrate concentrations (0, 0.15, 0.25, 0.5, 1, 2, 4, 8 and 10 mM) were used. CYP content per incubation was kept the same (40 pmol/mL) for all batches.

Furthermore, two separate sets of experiments were performed with 0.75 mM Cy in each of the three batches. Incubations were run as described above (15 min). One set was run with constant CYP concentrations, the other with the POR concentration kept constant (356 nmol cyt c reduced/min/mL, which is the same POR concentration as in batch 2 i.e. the batch with the median POR/CYP ratio), producing different concentrations of CYP and different POR/CYP ratios.

### 4-Hydroxy-cyclophosphamide preparation and detection

Maphosphamide was dissolved in phosphate buffer (50 mM, pH 7.4). The stock solution was added to plasma for the calibration curve for patient samples or further diluted with buffer to produce the standard curve for microsomal incubations. The produced 4-OH-Cy was stabilized and converted to a fluorescent dansylhydrazone derivative by adding dansylhydrazone (2 mg/mL) and one M hydrochloric acid. The mixture was vortexed and incubated at 50°C for 5 min. Thirty μL of the final sample was injected into the HPLC system which consisted of an LKB 2150 HPLC pump, a Gilson 234 auto-injector with a 100 μL sample loop and a Shimadzu RF-10XL Fluorescence detector. The detection was carried out with an excitation wavelength of 350 nm and an emission wavelength of 550 nm. The column was a Zorbax Extend C18, (150 x 4.6 mm, 5 μm microspheres) purchased from Agilent. The mobile phase was phosphate buffer (pH 3.5)/acetonitrile (2:1, v/v) and the flow rate was 1.4 mL/min. The retention time under these conditions was 13.4 min for 4 OH-Cy. The limit of quantitation was 100 pmol/mL and the calibration curve was linear between 60 and 2000 ng/mL [36, 37].

Data collection, chromatogram integration and determination of the 4-OH-Cy concentrations were carried out using a Clarity chromatographic station (version 2.8, DataApex Ltd., The Czech Republic) as integration software.

### Patients

For the detection of *POR* expression during Cy treatment, twenty patients undergoing HSCT at the Center for Allogeneic Stem Cell Transplantation (CAST), Karolinska University Hospital, Huddinge, Sweden, were included in the study. The study was approved by the ethical committee of Karolinska Institutet (number 616/03 and 2014/150-32). The complete data (ID no. 20051907) is available via the GEO database with the accession number “GSE51907“[38]. Written consent was obtained for all patients (for pediatric patients, consent was obtained by a legal guardian), and the individual(s) in this manuscript have given informed consent to publish these case details.

All patient characteristics including diagnosis, disease status at the time of transplantation, conditioning, age, type of transplantation and cell dose are shown in Table 2. Prior to transplantation, eleven patients were conditioned using 1 h i.v. Cy infusions (60 mg/kg, once daily for two days) followed by fractionated total body irradiation (fTBI). Nine patients were conditioned using fludarabine (Flu) 1 h i.v. infusions (30mg/m^2^/day, for 5 days) followed by 1 h i.v. Cy infusions (60 mg/kg, once daily for two days) and/or TBI. Blood samples were collected from each patient before and after Flu treatment and at the start of Cy infusion, 6 h after the first dose, before and 6 h after the second dose of Cy. For the determination of drug kinetics, both Cy and 4-OH-Cy were measured from patient plasma samples at several time points as described previously [36, 37, 39]. Due to technical problems, some samples were missed as reported in the complete data (ID no. 20051907).

**Table 2:** Patient characteristics.

Patient	Age (y)	Diagnosis	Conditioning regimen	CD 34 dose/kg	Disease status at HSCT	Outcome	Cause of death
**P 1**	57	B-CLL	Cy+fTBI (6 Gy)+ Alemtuzumab	14.7x10^6^	Transformed	† 10 months	Relapse
**P 2**	38	T-cell lymphoma	Cy+TBI	2.9x10^8^	PR	† 19 months	Relapse
**P 3**	31	AML	Cy+TBI+ATG	2x10^6^	CR2	† 6 months	Pneumonia
**P 4**	10	T-ALL	Cy+TBI+ATG	6.48x10^8^	CR1	† 12 months	Relapse
**P 5**	26	Pre-B ALL	Cy+TBI+ATG	13.5x10^6^	CR2	† 35 months	Relapse
**P 6**	19	ALL	Cy+TBI+ATG	13.5x10^6^	CR3	† 9 months	Relapse & pneumonia
**P 7**	51	AML	Cy+TBI+ATG	10.6x10^6^	Refractory	Alive 7.5 years	---
**P 8**	25	Pre–B ALL	Cy+TBI+ATG	7.3x10^6^	CR2	Alive 7.5 years	---
**P 9**	14	T-ALL	Cy+TBI+ATG	19.9x10^6^	CR2	Alive 7.2 years	---
**P 10**	41	T-cell lymphoma	Cy+TBI+ATG	9.3x10^6^	Relapse	† 51 days	Invasive fungal infection
**P 11**	26	T-ALL	Cy+TBI+ATG	0.5x10^5^0.2x10^5^	CR2	† 11 months	Relapse
**P 12**	49	NHL	Flu + Cy + TBI	4.0 x10^6^	PR	† 2 months	Relapse
**P 13**	11	Kidney Cancer	Flu + Cy + ATG	12.9 x10^6^	N/A	Alive 9 years	---
**P 14**	51	CLL	Flu + Cy + TBI + Campath	9.4 x10^6^	PR	† 2 months	Septic shock
**P 15**	57	NHL	Flu + Cy + TBI + ATG	6.1 x 10^6^	CR4	Alive 8.5 years	---
**P 16**	64	NHL	Flu + Cy + TBI + Campath	15.1 x10^6^	CR2	† 6 months	GI-bleeding
**P 17**	62	NHL	Flu + Cy + TBI	8.3 x10^6^	CR2	Alive 8 years	---
**P 18**	57	ALL	Cy + TBI + ATG	7.8 x10^6^	CR1	Alive 8 years	---
**P 19**	9	CLL	Flu + Cy + Campath	9.3 x10^6^	Relapse	† 6 months	Relapse & pneumonia
**P 20**	9	CLL	Flu + Cy + TBI	11.0 x10^6^	PR	† 8 months	Relapse, pneumonia & septicemia

**Abbreviations:** ALL: acute lymphoblastic leukemia; AML: acute myeloid leukemia; ATG: antithymocyte globulin; B: B lymphocyte; CLL: chronic lymphoblastic leukemia; CD 34: bone marrow-derived stem cells; CR: complete remission; Cy: cyclophosphamide; Flu: Fludarabine; HSCT: hematopoietic stem cell transplantation; NHL: Non-Hodgkin lymphoma; P: patient; PR: partial remission; T: T lymphocyte; TBI: Total body irradiation;

^†^: survival time. fTBI: fractionated total body irradiation

Patient mRNA was subjected to analysis of global gene expression by NimbleGen microarrays (Roche Diagnostics Scandinavia, Bromma, Sweden). Data were analyzed using GeneSpring GX (Agilent, CA, USA). The expression data were normalized using quantile normalization and the gene expression data were generated using the Robust Multichip Average algorithm. Significant differences in gene expression were determined by ANOVA. The selection threshold of a false discovery rate (FDR) was <5% and the fold change in the SAM output result was >2. RNA extraction, cDNA preparation and gene array analysis were performed as described previously [38].

### PCR

Samples were scanned using TaqMan genotyping PCR primer for the only polymorphism reported to increase CYP activity *in vivo*, POR*28 (catalogue #4362691, SNP rs1057868C>T, Applied Biosystems, Stockholm, Sweden). Samples were extracted (catalogue # 51304, Qiagen, Stockholm, Sweden) and amplified, according to the manufacturer’s instructions, in 72 rotor (20 μL total volume) containing 1x TaqMan SNP Genotyping master mix (catalogue # 208252, Qiagen, Stockholm, Sweden). Analysis was performed using Rotor Gene Real-Time PCR System (Qiagen, Stockholm, Sweden) by means of the VIC and FAM dye-labeling system according to the manufacturer’s instructions. Genotypes were assigned post-PCR end-point reading, using the manual calling option in the allelic discrimination application. Negative controls were run in parallel to the samples.

### Data Analysis

Calculations, statistics and graphs were performed using GraphPad Prism (version 4.0, GraphPad Software, Inc.) and SigmaPlot (version 12.5, Systat software,Inc.). Prior to all analysis, all patient data were tested and approved to be normally distributed.

## Results

### *In vitro* cyclophosphamide metabolism

The linearity of Cy metabolism was studied with both time and CYP concentration, and the 4-OH-Cy formation was found to be linear up to 1 mM Cy and at CYP concentrations between 10 and 40 pmol/mL and incubation times up to 25 min.

4-OH-Cy produced from maphosphamide has a purity of 99.9% according to the manufacturer. Hydroxylation of maphosphamide in aqueous solution and/or plasma yielded 4-OH-Cy and cyclohexylamine. Cyclohexylamine did not interfere with the fluorescence detection of 4-OH-Cy.

**Fig 1:**
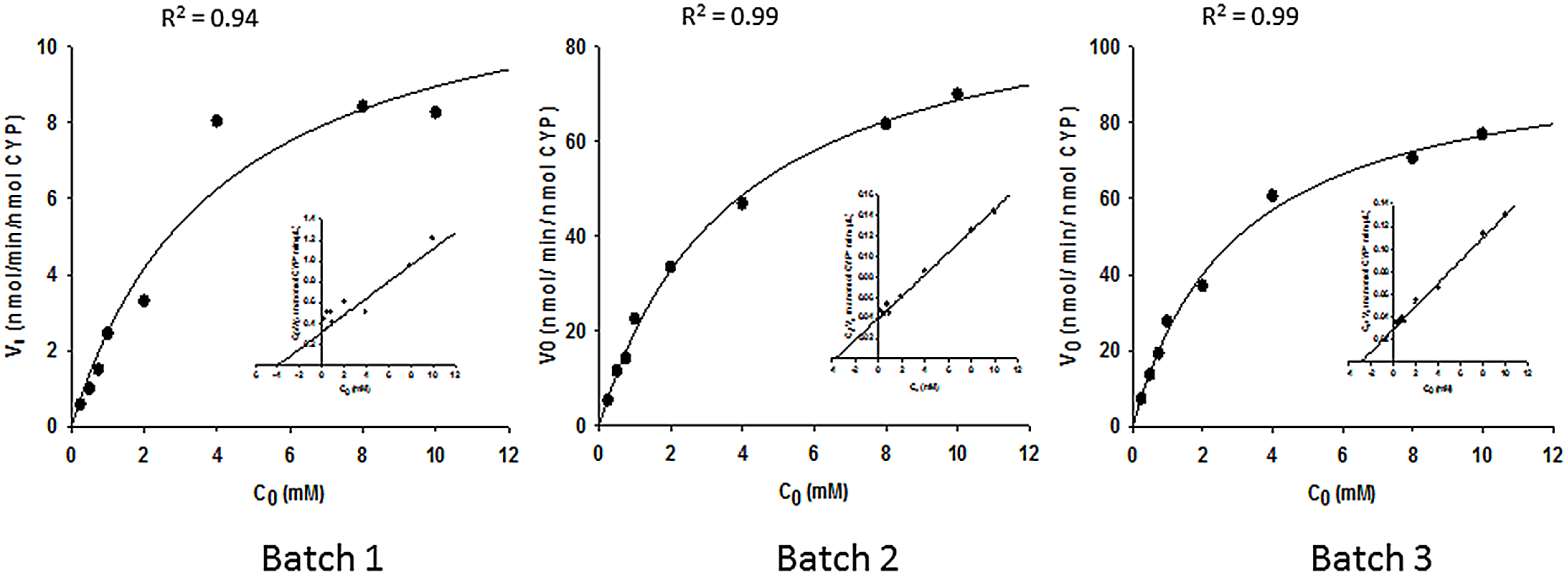
Michaelis–Menten curves and Hanes-Woolf plots for 4-OH-Cyclophosphamide enzyme kinetics for different batches of CYP2B6.1. In 2 independent experiments, Cyclophosphamide (at different concentrations) was incubated with 3 batches of *Escherichia coli* microsomes containing cDNA-expressed CYP2B6.1 (See Table 1) with different POR/CYP ratios. The CYP concentration per mg protein and the protein concentration per mL incubation were similar between batches. The 4-OH-Cy formation was measured using HPLC with fluorescence detector. The results shown are the averages obtained with each of the three batches. Fitting the data to Michaelis-Menten kinetics gave an apparent intrinsic clearance, *CL*_*int*_ (*V*_max_/*K*_m_), of 3.1, 25.1, 33.7 μL/min/nmol CYP for Batch 1, Batch 2 and Batch 3, respectively. The intrinsic clearance thus increased with increasing POR/CYP ratio. See also Fig 2 and Table 3.

The enzyme kinetics for 4-OH-Cy formation with microsomes containing human cDNA-expressed CYP2B6.1 and various ratios of POR/CYP was determined under linear conditions. Fitting the data from two independent experiments (each with the three different batches of microsomes at a constant concentration of 40 pmol CYP/mL and Cy concentration up to 10 mM) to Michaelis-Menten kinetics, the apparent *K*_*m*_ for CYP2B6.1 was similar for the 3 batches (3–4 mM). However, the apparent maximum rate of Cy 4-hydroxylation (*V*_*max*_) increased with the POR/CYP ratio, ranging from 12.6 to 99.0 nmol/min/nmol CYP (Fig 1). The CYP concentrations and the protein concentrations were almost identical in the different batches, indicating that the the electron transfer by POR from NADPH to CYP may be rate limiting and that the POR/CYP ratio thus may be important for the Cy kinetics *in vitro*.

**Fig 2:**
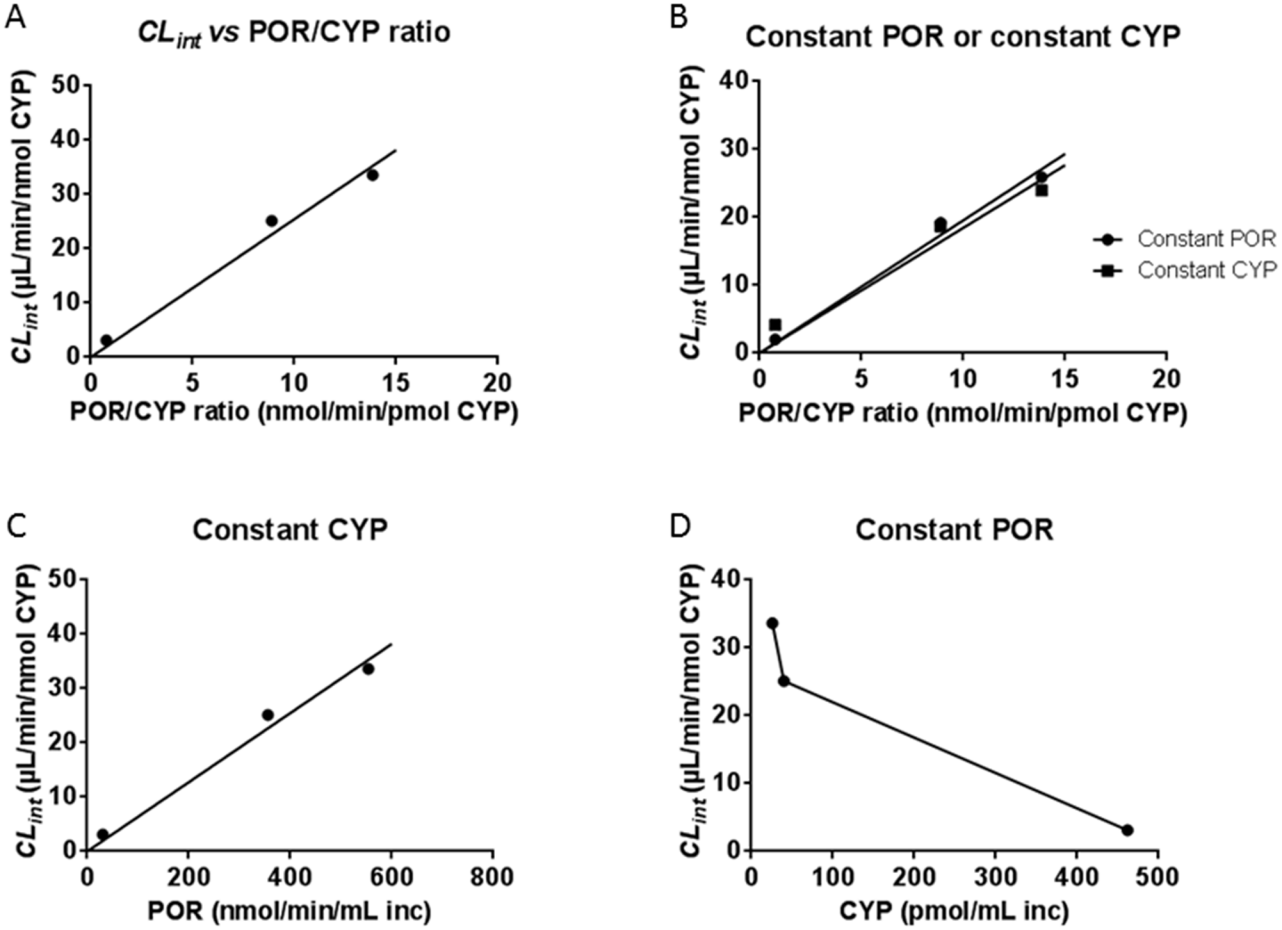
Cyclophosphamide intrinsic clearance for different CYP2B6.1 batches. Cy (0.75 mM) was incubated with different batches (Batch 1–3, Table 1) of microsomes containing CYP2B6.1. In Fig 2A, the intrinsic clearance, *CL*_*int*_, or ratio of *V*_*max*_/*K*_*m*_ (3.1–33.7 μL/min/nmol CYP) determined from the Michaelis-Menten kinetics was found to be proportional to the POR/CYP ratio (0.77–13.8 nmol/min/pmol CYP). In Fig 2B, the *CL*_*int*_ approximated from the *v*_*0*_ */C*_*0*_ in experiments with either constant CYP (40 pmol/mL incubation) or constant POR (356 nmol/min/mL incubation) is plotted for the 3 batches. The *CL*_*int*_ values are shown to increase proportionally with the POR/CYP ratio regardless of if POR or CYP is kept constant. With constant CYP, the *CL*_*int*_ was found to be proportional to the POR concentration (Fig 2C) while it did not increase with CYP content in experiments with low, constant concentrations of POR (Fig 2D).

This also resulted in the intrinsic clearance, *CL*_*int*_ (i.e. the ratio of *V*_*max*_/*K*_*m*_) being proportional to the POR/CYP ratio ranging between 3.1 and 33.7 μL/min/nmol CYP (Fig 2A and Table 3).

**Table 3:** Apparent enzyme kinetic parameters for 4-OH-Cy formation by different microsome batches with recombinant CYP2B6*1 and different POR/CYP ratios. Cyclophosphamide at different concentrations was incubated in 2 independent experiments with 3 batches of CYP2B6.1 containing different POR/CYP ratios. The 4-OH-Cy formation, used as a measure of Cy metabolism, was measured using HPLC. The results shown are the averages obtained for each of the three batches. The results show almost proportional increase of Cy intrinsic clearance (i.e. apparent *V*_*max*_/*K*_*m*_, with *V*_*max*_ measured from 4-OH-Cy formation) with higher POR/CYP ratios from Batch 1 to Batch 3.

Enzyme kinetic parameter	Batch 1	Batch 2	Batch 3
**POR** * **/CYP (nmol/min/pmol CYP)**	0.77	8.90	13.85
***K***_***m***_ **(mM Cy)**	4.02	3.76	2.94
***V***_***max***_ ** **(nmol/min/ nmol CYP)**	12.55	94.54	99.09
***CL***_***int***_ **(μl/min/ nmol CYP)**	3.12	25.13	33.66

* POR measured as Cytochrome c reductase activity as in Table 1

** *V*_*max*_ is based on 4-OH-Cy formed

***CL***_***int***_ Calculated as ***V***_***max***_ ***/K***_***m***_

To further study the effect of different POR/CYP ratio in the three batches of CYP2B6.1, two separate sets of incubations were performed for 15 minutes with 0.75 mM Cy and each of the three batches. Experiments were performed with constant CYP amounts (40 pmol/mL) resulting in different POR concentrations (Fig 2B and 2C). Separate experiments were performed with constant POR activities (356 nmol cyt c reduced /min/mL) resulting in different CYP concentrations (Fig 2B and 2D). The *CL*_*int*_ values were approximated from the *v*_*0*_ */C*_*0*_ and they correlated (R^2^ = 0.99) with the POR/CYP ratios (regardless of if POR or CYP concentrations were kept constant). The *CL*_*int*_ values are shown to increase proportionally with the POR/CYP ratio regardless of if POR or CYP is kept constant. With constant CYP, the *CL*_*int*_ was found to be proportional to the POR concentration (Fig 2C) while it did not increase with CYP content in experiments with low, constant concentrations of POR (Fig 2D) and an excess of CYP per POR. Thus, all experiments showed a strong correlation between the POR/CYP ratio and the Cy 4-hydroxylase activity, confirming that the POR/CYP ratio will affect the Cy kinetics, at least *in vitro*.

In microsomes containing POR only, no 4-OH-Cy formation could be detected, indicating that POR, by itself, could not bioactivate Cy.

### Patients

The clinical outcome and survival time for the patients that participated in the study is listed in Table 2. As shown, the majority of patients died due to relapse or infection.

**Fig 3:**
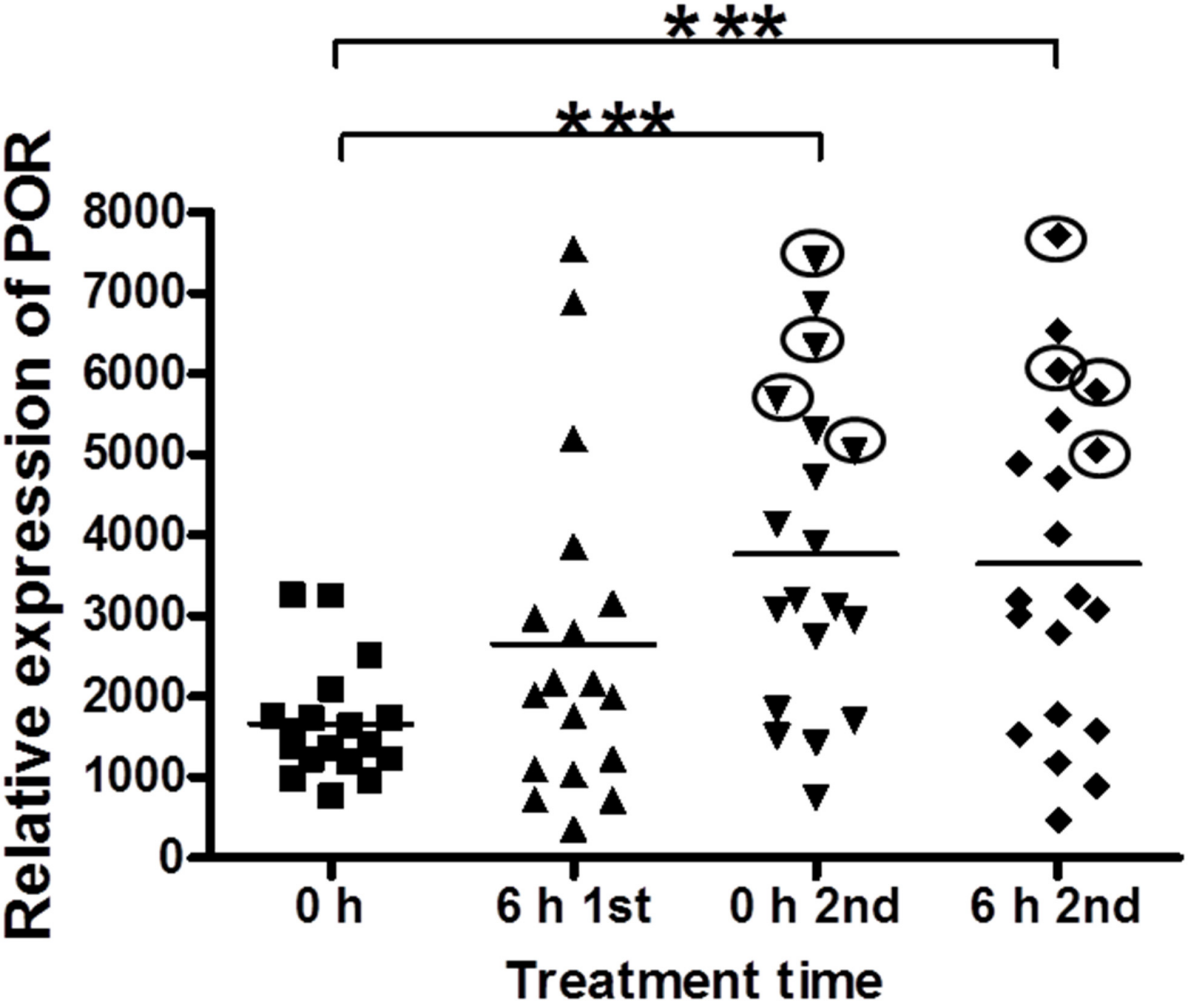
Up-regulation of *POR* mRNA during Cy conditioning. The gene expression of *POR* was significantly (*p* < 0.001, t-test) up-regulated 24 hours after the first Cy infusion and 6 hours after the second dose of Cy, compared to pre-Cy conditioning. The inter-individual variation in relative POR expression increased from 4.3-fold before Cy conditioning to 9.9-fold before the 2^nd^ dose of Cy and to 16.5-fold by the end of the treatment. The high variation in up-regulation of *POR* gene expression during Cy treatment may contribute to the high inter-individual variations in Cy kinetics reported in patients, possibly in part due to different inducibility of the polymorphic forms of *POR*. Circles in the figure are for four patients with POR*28 polymorphism.

The average expression level of the *POR* gene was significantly (*p* <0.001, t-test) up-regulated 24 h and 30 h after the first Cy infusion (i.e. before and 6 h after the second infusion), compared to the levels before conditioning. However, a high inter-individual variation in *POR* gene expression after Cy infusion was observed, with a difference in individual relative POR expression of 4.3-fold before Cy conditioning increasing to 9.9-fold after 24 h and 16.5-fold by the end of the treatment, i.e. the expression levels increased in some patients more than others. The average expression levels after Cy treatment increased by 2.2 fold compared to the average before the start of the treatment (Fig 3).

**Fig 4:**
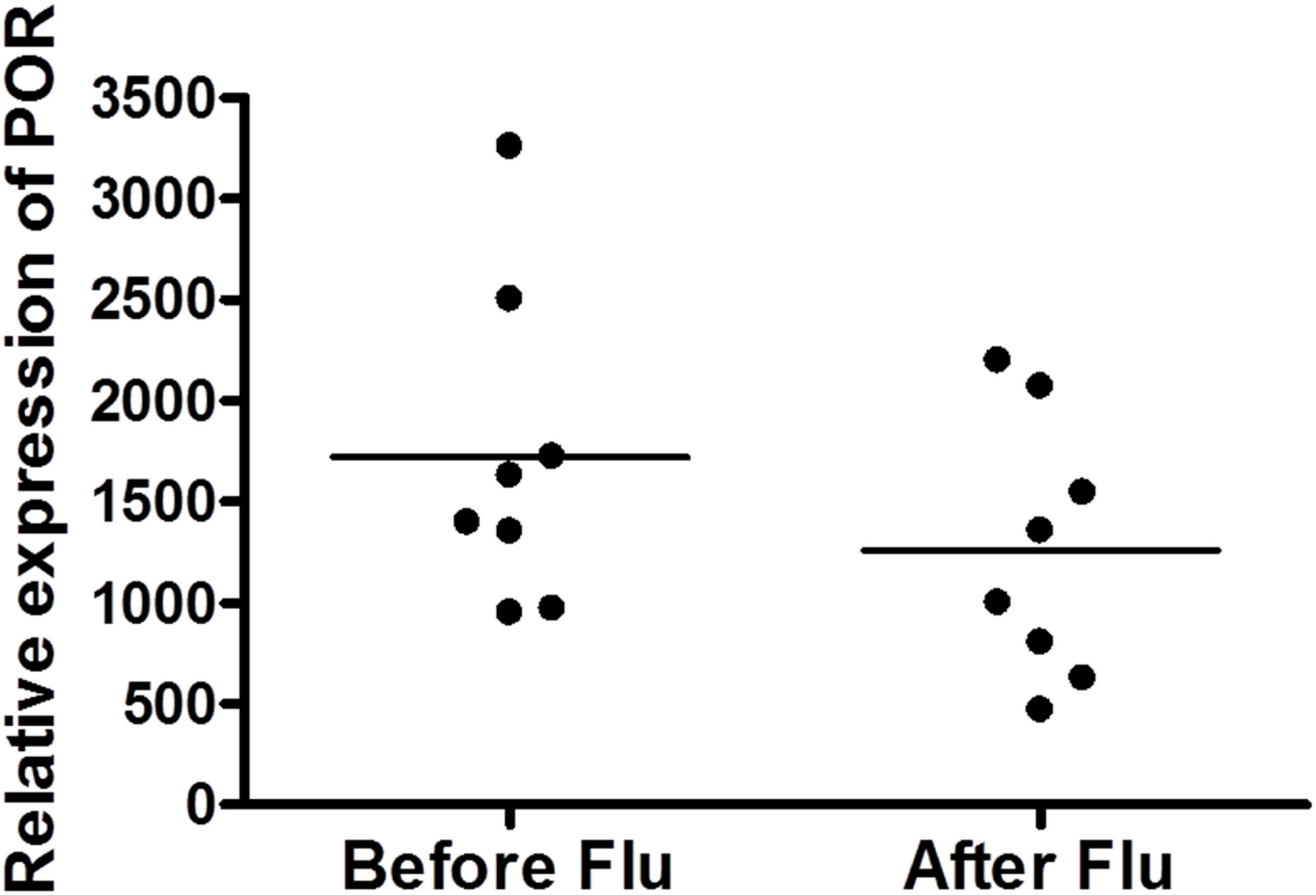
Effect of fludarabine on *POR* gene expression. The gene expression of *POR* was not significantly changed after treatment with fludarabine.

For the patients treated with fludarabine before Cy, the increase in *POR* expression after treatment with Cy was similar to that found in patients treated with Cy. Moreover, no significant change was found in *POR* expression after fludarabine treatment and prior to Cy (Fig 4).

**Fig 5:**
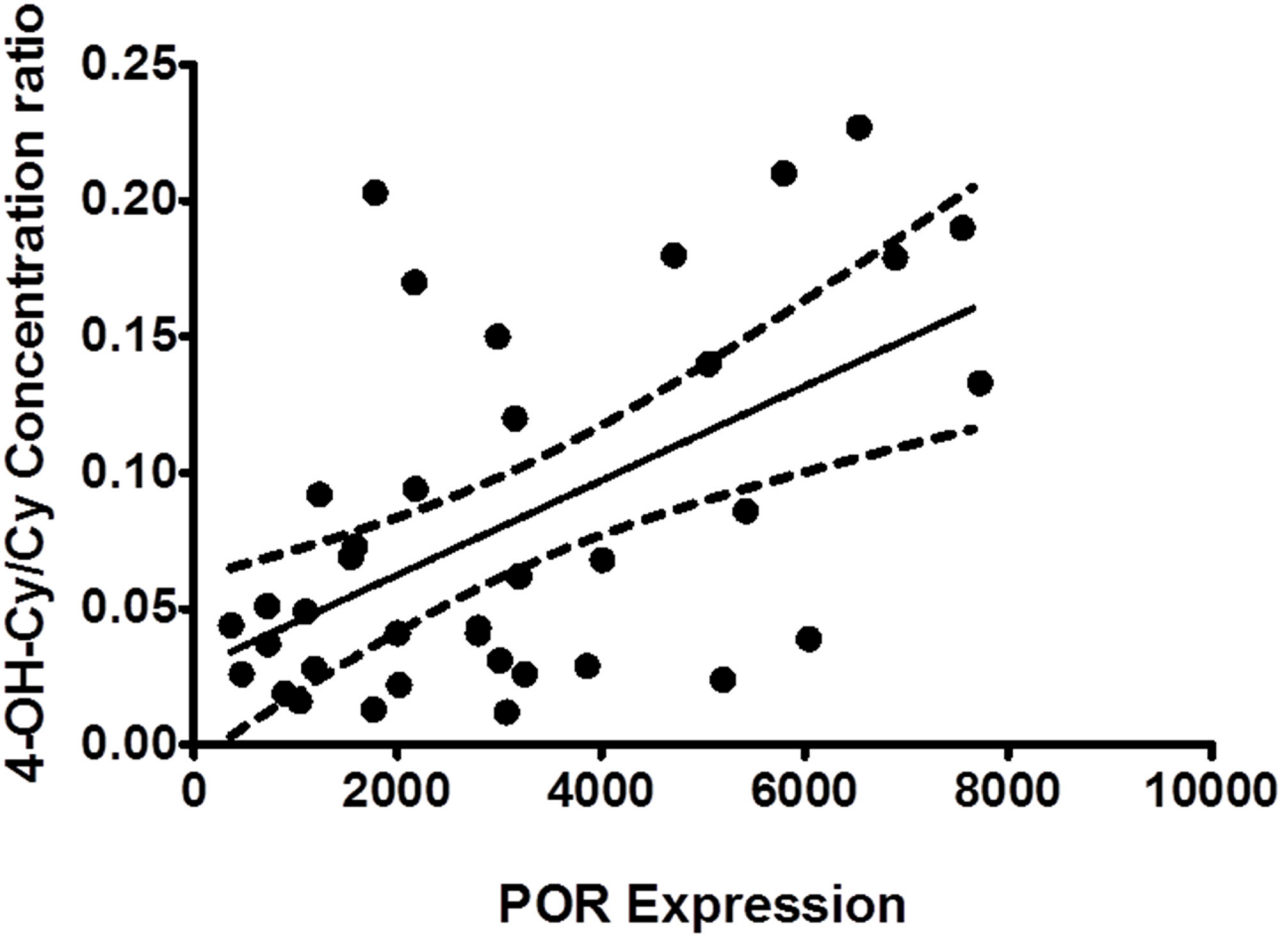
The correlation between *POR* expression and 4-OH-Cy/Cy concentration ratio. The concentration ratio for 4-OH-Cy/Cy (as measure for the bioactivation of Cy) in plasma at 6 and 24 h (0 time before next dose) after first dose and at 6 h after the second dose was significantly (P < 0.001, r2 = 0.308, linear regression) correlated with *POR* expression in patients (*in vivo)*.

The concentration ratio (4-OH-Cy/Cy) at 6 h after first dose and at 6 h after the second dose was significantly (*P*<0.001, r^2^ = 0.308, linear regression) correlated with *POR* expression as shown in Fig 5.

PCR results for POR*28 showed that five patients carried the homozygous mutation and four patients carried the heterozygous mutation. The rest of the patients did not carry this mutation. Four out of the patients carrying POR*28 mutations had relatively high *POR* expression 24 h and 30 h after the first Cy infusion. However, four other patients carrying no POR*28 mutation also had the same high *POR* expression. No-template controls (NTC, n = 10) served as negative controls to ascertain that there was no contamination of reagents and plates.

## Discussion

Genetic polymorphism has been associated with variable levels of gene expression in the liver, and CYP2B6 appears to be one of the most polymorphic human P450s.

Human CYP2B6 expression levels for the C1459T mutation (alleles *5 and *7) have been reported to be significantly lower than for CYP2B6*1 [12], while this mutation leads to a higher Cy intrinsic clearance both *in vitro* and *in vivo* [8, 15]. In Caucasians, the SNP frequency has been reported to be 33% and 29% for A785G and G516T, respectively. [12]. These SNPs are present in several *CYP2B6* allelic variants such as (2B6*4, 2B6*6, 2B6*7 and 2B6*9).

Ariyoshi *et al*. have reported higher activity of CYP2B6*6 in 4-hydroxylation of Cy and decreased activity of 8-hydroxylation of efavirenz metabolism. The authors used microsomes containing CYP2B6.1, CYP2B6.4 or CYP2B6.6, but with the same POR activity and POR/CYP ratio in each batch for their *in vitro* experiments [40].

On the other hand, Raccor *et al*. have reported that *CYP2B6* genotype is not related to 4-OH-Cy formation either *in vitro* or *in vivo* [18]. Moreover, other studies have shown that the genotype of *CYP2B6*, or other CYPs involved in Cy metabolism, do not affect Cy kinetics and suggest that clinical factors such as patient age and cancer grade may be more significant [17, 19, 20].

POR is the main electron donor for all microsomal CYP monooxygenases and its polymorphisms have been shown to affect CYP-mediated drug metabolism as well as direct bioactivation of prodrugs [41]. Chen *et al*. studied the effect of POR genotype on CYP2B6-dependent bupropion metabolism. Their results showed a 70–74% reduction in CYP2B6 activity with some *POR* polymorphisms *in vitro* [31]. That was in agreement with another study, which also reported that *S*-Mephenytoin *N*-demethylation by CYP2B6 varied with the POR polymorphism in the human liver [32]. CYP-dependent drug metabolism, like Cy 4-hydroxylation, depends on POR for the electron transfer from NADPH to CYP. *POR* expression levels and its electron transfer efficiency may be rate limiting for fast reactions and could affect drug kinetics, overall exposure and metabolic pattern and hence affect efficacy and side effects.

The formation of 4-OH-Cy is a fast reaction, which means that the rate limiting step may be the electron transfer from the POR to CYP [41]. In an experiment with different batches of recombinant human CYP2B6.1, the intrinsic clearance of Cy was clearly proportional to the POR/CYP ratio ranging between 3.1 and 33.7 μL/min/nmol CYP despite the fact that *K*_*m*_ was almost constant in all batches. This experiment was performed once with constant CYP in all incubations and then repeated with constant POR in all incubations with the same batches. The results showed good linear correlation between 4-OH Cy formation and POR/CYP ratio.

POR variability also affects CYPs other than CYP2B6, such as CYP2C9 activities, when incubated with flurbiprofen, diclofenac, and tolbutamide. These drugs, like Cy, are metabolized rapidly [29]. The effect of POR variants and expression levels varies with the substrate and the CYP enzyme variant; for example, *POR* polymorphic variants with A287P and/or R457H are associated with no detectable CYP2D6 metabolism of 7-ethoxymethoxy- 3- Cyanocoumarin (EOMCC), while Q153R polymorphism increased CYP2D6 activity with EOMCC *in vitro* [28].

Steroidogenic activity is dependent mainly on CYP1A2 and CYP2C19. POR variants affected the activities of these enzymes to different extents. *POR* polymorphisms A287P and/or R457H have reduced CYP1A2 and CYP2C19 catalytic activities to EOMCC. The A503V polymorphism gave 85% of wild-type activity with CYP1A2 and 113% of wild-type activity with CYP2C19, while Q153R polymorphism increased both CYP1A2 and CYP2C19 activities [27].

CYP3A4 completely lost its capacity for 6-beta-hydroxylation of testosterone *in vitro* by two of the *POR* polymorphisms, Y181D and A287P. Other *POR* polymorphisms, such as K49N, A115V and G413S, resulted in increased CYP3A4 activity for testosterone with up to 65% [31]. Tacrolimus is metabolized by CYP3A5 and the POR*28 polymorphism was shown to result in a significant decrease in tacrolimus exposure. POR*28 along with the *CYP3A5* polymorphism was responsible for the inter-individual variability of tacrolimus [30].

A study on human liver samples showed that four *POR* polymorphisms (K49N, L420M, A503V, and L577P) have both reduced POR expression levels and drug-metabolizing CYP activity. The same study also showed that intron polymorphisms altered POR activity [41].

CYP2B6 polymorphism can affect the drug kinetics. In the present study, however, the influence of POR was studied for only one CYP2B6 variant (CYP2B6.1). The relative amount of POR significantly influenced the drug metabolic activity.

Results obtained from *POR* gene expression measurements in samples from 20 patients conditioned with Cy showed significant (*p*<0.001) *POR* up-regulation at 24 h and 30 h after the first Cy infusion (i.e. before and 6 h after the second Cy infusion). However, high inter-individual variations in gene expression before and after Cy infusion was observed. *POR* expression showed significant positive correlation with the concentration ratio 4-OH-Cy/Cy (as measure for the bioactivation of Cy), which may explain part of the inter-individual variation in Cy kinetics.

Although half of the patients had been treated with Flu before Cy, Flu didn’t show any effect on the *POR* up-regulation. Flu is a prodrug which is converted to its active metabolite through a process of dephosphorylation and phosphorylation, neither of which need any of the CYPs or POR [42].

PCR results for POR*28 genotype showed that almost half of the patients were carriers for POR*28. Five of them had significantly higher *POR* expression after Cy conditioning. However, four other patients also had high *POR* expression, possibly due to other *POR* polymorphisms not yet described, or to effects on nuclear receptors or other factors involved in *POR* regulation, also resulting in higher inducibility.

Determination of patient genotype and expression levels for *POR* [43] as well as *CYP2B6* before Cy therapy, may be utilized for personalizing treatment. Moreover, it might help make the clinical decision for concomitant treatment with other drugs that are metabolized by these enzymes. This may improve Cy treatment, decrease its adverse effect and hence improve the clinical outcome. More studies are warranted to clarify the role of POR in Cy metabolism.

## Conclusions

The present results indicate that not only CYPs are important, but also *POR* expression and/or activity may influence Cy bioactivation affecting treatment efficacy. Thus, both POR and CYP genotype and expression levels may have to be taken into account when personalizing treatment schedules to achieve optimal therapeutic drug plasma concentrations.
